# Real-world evaluation of prevalence, cohort characteristics, and healthcare utilization and expenditures among adults and children with autism spectrum disorder, attention-deficit hyperactivity disorder, or both

**DOI:** 10.1186/s12913-025-13296-2

**Published:** 2025-08-09

**Authors:** Amanda L. Zaleski, Kelly J. Thomas Craig, Raiha Khan, Rebecca Waber, Wei Xin, Makia Powers, Una Ramey, Dorothea J. Verbrugge, Deborah Fernandez-Turner

**Affiliations:** 1 Clinical Evidence Development, CVS Health ®, Wellesley, MA USA; 2 Analytics and Behavior Change, CVS Health, Wellesley, MA USA; 3 Commercial Product & Innovation, CVS Health, Wellesley, MA USA; 4 Aetna Medical Affairs, CVS Health, Wellesley, MA USA; 5 Clinical Evidence Development Aetna ® Medical Affairs, CVS Health ®, 151 Farmington Avenue, 06156 Hartford, CT USA

**Keywords:** Neurodevelopmental, Behavioral health, Comorbid conditions, Economic evaluation, Neurodiversity

## Abstract

**Background:**

Autism spectrum disorder (ASD) and attention-deficit/hyperactivity disorder (ADHD) are among the most common neurodevelopmental disorders. However, significant gaps persist in understanding health and healthcare-related needs of individuals diagnosed with ASD and/or ADHD across the lifespan. Thus, this real-world evaluation sought to characterize the prevalence of ASD, ADHD, and co-existing ASD and ADHD (AuDHD); sociodemographics; frequent comorbidities and co-occurring diagnoses; and healthcare utilization and expenditures among members of a large national payor.

**Methods:**

This study represents an observational, cross-sectional evaluation of claims from a large national payor. Retrospective claims analyses of commercial fully insured (C-FI) members from 1/1/2022–12/31/2022 identified diagnoses for ASD and/or ADHD among adults (≥ 18 year) and children (< 18 year). Chi-squared tests, *T*-tests, and Fisher’s exact tests examined between-group differences in sociodemographic, health, and healthcare-related measures among members with neurodevelopmental disorders compared to members without ASD and/or ADHD.

**Results:**

Within adults (*N* = 1,928,106), 4.2% of members (60.2% White, 52.9% female, mean age: 34.1 ± 10.9 year) were diagnosed with neurodevelopmental disorders: ADHD (4.0%, *n* = 76,515); ASD (0.1%, *n* = 2,134); or AuDHD (0.1%, *n* = 1,266) (all *P* < 0.0001). Within children (*N* = 464,749), 6.7% of members (47.8% White, 67.5% male, mean age: 11.3 ± 3.8 year) were diagnosed with neurodevelopmental disorders: ADHD (5.0%, *n* = 23,250); ASD (1.1%, *n* = 5,098); or AuDHD (0.6%, *n* = 2,665) (all *P* < 0.0001). Increased odds (i.e., ≥ 2) for certain co-occurring diagnoses were consistently observed across all three neurodevelopmental cohorts for adults and children, which were primarily behavioral health (BH)-related. Compared to those without neurodevelopmental disorders, both adults and children with ASD and/or ADHD had higher healthcare utilization rates [adults: 615.2 to 1024.8 per thousand per month (PTPM); children: 398.4 to 1205.3 PTPM; all *P* < 0.001)]; largely owing to increased use of BH-related services, translating to greater total healthcare expenditures [adults: $140.3 to $292.1 per member per month (PMPM); children: $50.8 to $845.4 PMPM; all *P* < 0.001)].

**Conclusions:**

Leveraging real-world data of 2,392,855 members from a large national payor, 4.1% of adults and 6.7% of children were diagnosed with ASD and/or ADHD. This population appeared to consistently exhibit specific co-existing diagnoses that frequently co-occur in addition to greater observed healthcare utilization and expenditures.

*Trial registration* Not applicable.

**Supplementary Information:**

The online version contains supplementary material available at 10.1186/s12913-025-13296-2.

## Introduction

Autism spectrum disorder (ASD) and attention-deficit/hyperactivity disorder (ADHD) are among the most common neurodevelopmental disorders in the United States (US) and world. ASD is characterized by differences in social communication and interactions, and the presence of restricted and repetitive interests, behaviors, and/or activities [1], with an estimated prevalence of ~ 1–3.2% among US children ages 4–8 years old [2, 3] and 2.2% among US adults [4]. ADHD, which manifests as persistent patterns of inattention, hyperactivity, and impulsivity that interferes with daily functioning [1], affects 11% of US children ages 5–17 years old [5] and approximately 3% of adults worldwide [6]. It has long been observed that ASD and ADHD commonly co-occur in children [7, 8] and adults [9]; however, true estimates of co-occurrence vary widely and are poorly understood. This is partly because ASD and ADHD were not formally recognized as co-occurring conditions until the publication of the Diagnostic and Statistical Manual of Mental Disorders, Fifth Edition in 2013 [10], which allowed for dual diagnosis. In addition, the US has yet to establish a surveillance system to collect population-based data of adults living with ASD [4]. Consequently, research on individuals with co-existing ASD and ADHD, particularly among adults, is still in its infancy, necessitating a more nuanced understanding of shared needs associated with these disorders [11, 12].

Indeed, a growing body of evidence suggests a strong association between neurodevelopmental disorders and specific mental health comorbidities, such as depression and anxiety in children [13], adolescents [13], and adults [14]. These comorbidities often complicate the diagnosis and treatment of ASD and/or ADHD, resulting in decreased quality of life and increased healthcare utilization and expenditures [15]. Furthermore, significant disparities for historically marginalized groups on the basis of age, sex, gender, race, ethnicity, and socioeconomic status exist across the care continuum [16] that are less likely to receive timely diagnosis and appropriate care [17–21]. These disparities further exacerbate health and healthcare challenges already faced by individuals with ASD and/or ADHD [22], contributing to broader health inequities.

Lastly, the growing recognition of neurodiversity (i.e., natural variation in the human brain that leads to differences in how we think and behave) within the workforce has led to increasing employer demand for better support structures for adults with ASD and/or ADHD and beyond [23, 24]. However, effective implementation of inclusion and belonging workplace practices first require a comprehensive understanding of unique healthcare needs. Current research has primarily focused on pediatric populations and there remains a substantial gap in understanding the prevalence, healthcare needs, and comorbidities of adults with neurodevelopmental disorders across the entire lifespan [25–28]. Given that ASD and/or ADHD often co-exist with other mental and physical health conditions, it is crucial to characterize the broader health burden carried by individuals with these diagnoses.

Taken altogether, there is a critical need for rigorous, comprehensive research that extends beyond pediatric-centric perspectives to include individuals with ASD, ADHD, and co-existing ASD and ADHD across the lifespan. The use of real-world data, particularly claims-based data, offers a valuable opportunity to examine health- and healthcare-related trends of individuals with neurodevelopmental disorders on a population level. As such, this real-world evaluation aimed to characterize the prevalence, sociodemographic composition, and chronic comorbidities of adults and children diagnosed with ASD and/or ADHD within a large national health plan. As a secondary aim, differences in healthcare utilization and related expenditures between members with and without these neurodevelopmental disorders were also explored. We hypothesized that members with ASD and/or ADHD would be more likely to have more co-existing diagnoses and comorbidities and higher rates of healthcare utilization and expenditures compared to members without these disorders.

## Methods

### Study design

This study deployed an observational cohort design. Briefly, retrospective claims analyses of commercial fully insured (C-FI) members using the 10th revision of the International Classification of Diseases (ICD-10) identified members with neurodevelopmental diagnoses for ASD (F84.0) and ADHD (F90.X) and informed the formation of three neurodevelopmental cohorts: (a) ASD, (b) ADHD, or (c) co-existing ASD and ADHD (referred to as “*AuDHD”* hereafter). Neurodevelopmental cohorts were then compared to a comparison cohort, defined as members without a diagnosis for ASD or ADHD. Analysis of de-identified, aggregated data characterized the (1) point prevalence of ASD and/or ADHD; (2) sociodemographics; (3) point prevalence and odds of comorbidities and co-occurring diagnoses; (4) healthcare utilization rates; and (5) healthcare expenditures for each neurodevelopmental and age cohort.

### Study population

All participants were C-FI enrollees of a large national health plan. Members were included for evaluation if they had ≥ 1 month (mo) of continuous medical benefit coverage during the evaluation period (i.e., January 1, 2022, to December 31, 2022). Members were excluded if they did not meet inclusion criteria. The neurodevelopmental cohort included members with presence of ≥ 1 ICD-10 code for ASD and/or ADHD. The comparison cohort included members without an ICD-10 code for ASD and/or ADHD.

### Data sources and study setting

All demographic and administrative medical claims data were de-identified, aggregated, and analyzed. Demographic information collected during health insurance enrollment included self-reported sex, age, race, ethnicity, location, plan benefit details, and census tract statistics. Claims data included diagnosis codes, procedures, laboratory encounters, sites of care, provider information, and service costs. Claims data also included aggregations of the above information in the forms of medical cases, chronic condition flags, and predictive risk scores. The Aetna Integrated Informatics^SM^ Health Profile Database (HPD) was used to identify co-existing chronic comorbidities and diagnoses for each neurodevelopmental and age cohort. Briefly, the HPD database is comprised of > 90 chronic diseases or medical conditions identified through rule-based logic applied to structured administrative data sources. The proprietary algorithm is constructed using well-established and validated sources commonly employed in clinical, operational, and epidemiologic research, including medical claims, pharmacy claims, and clinical laboratory data from physician encounters, specialist visits, facilities, and laboratories, and others. Condition identification is based on hierarchical groupings of standardized code sets such as ICD-10 diagnosis codes and Current Procedure Terminology 4th revision (CPT-4) procedure codes. In general, standardized inclusion and exclusion logic are used to identify disease categories. For operational reporting and prevalence tracking, a single-criteria or ‘one hit’ method is deployed based on at least one occurrence of any of the specific diagnosis criteria. For research purposes, a more stringent identification criterion is applied. Specifically, members were flagged as having a condition only if diagnosis criteria were met in two of five source systems or with ≥ 3 qualifying events (e.g., diagnosis, procedure, laboratory, or pharmacy claim). This rule-based, deterministic approach enhances identification sensitivity and consistency through multisource triangulation and hierarchical code classification. The database is refreshed monthly and includes all dates of service within an 18-month timeframe.

### Statistical analyses

All data were de-identified, aggregated, and analyzed. Demographic and administrative claims data were used to calculate prevalence of ASD and/or ADHD (i.e., frequency and %) and sociodemographic characteristics (i.e., % age, gender, race, ethnicity, and geography) of the total evaluated population. All instances of missing data were addressed using descriptive methods. For self-reported variables including race, ethnicity, and gender, a ‘missing or unknown’ category was incorporated to ensure the inclusion of all participants in the analysis and to minimize the risk of selection bias. No additional missing data were observed, as the remaining sociodemographic variables (i.e., geographic location and date of birth) are mandatory fields collected at the time of plan enrollment.

Claims data were also used to compute average total healthcare utilization rates and reported as per thousand per month (PTPM) ± 95% confidence interval (CI). Healthcare utilization rates were also segmented into the following sub-categories: emergency department (ED), primary care provider (PCP), and specialist visits. These sub-categories were further stratified by behavioral health (BH)-related visits, including BH-related ED, BH-related PCP, and BH-related specialist visits. Non-BH-related and BH-related subcategories were treated as mutually exclusive. BH-related visits were classified using a service classification system derived from medical cost categories and their associated subcategory codes. This operational definition captured BH-related visits across inpatient, emergency, outpatient, and office-based settings using BH-specific groupings. Classification was based on claim-level information including procedure codes, service categories, and provider specialty. Examples of BH-related claims include behavioral health counseling and therapy, psychotherapy provided by a non-mental health specialist, and substance abuse-related ED visits. This logic was developed through internal clinical expertise and is applied consistently across research and operational settings.

Difference in healthcare utilization rates were calculated as healthcare utilization for the neurodevelopmental cohort minus healthcare utilization for the comparison cohort, such that positive values indicate higher utilization for members with ASD and/or ADHD compared to members without ASD and/or ADHD. Utilization outcomes were annualized and reported on a per member basis to account for variation in plan enrollment duration.

Similarly, claims data were also used to compute average healthcare expenditures in US dollars (USD, proprietary data not shown). Healthcare expenditures were segmented into the previously outlined medical cost sub-categories and reported as spend for per member per month (PMPM) ± 95% CI. Difference in healthcare expenditures was calculated as healthcare spend for the neurodevelopmental cohort minus healthcare expenditures for the comparison cohort, such that positive values indicate higher spend for members with ASD and/or ADHD compared to members without ASD and/or ADHD. Healthcare utilization and expenditures were capped at the 99th percentile. Expenditures were annualized and reported on a per member basis to account for variation in plan enrollment duration.

The HPD database was used to characterize co-existing chronic comorbidities and diagnoses for each neurodevelopmental and age cohort. Fisher exact tests calculated odds ratio (OR) values to indicate the strength of association between neurodevelopmental cohorts and co-existing diagnoses. All statistically significant OR values ≥ 2 were reported to prioritize findings for interpretation across a large number of comparisons. This threshold indicates a moderate-to-strong effect size and is commonly applied in observational and epidemiologic studies to highlight associations that may be clinically meaningful and actionable [29, 30].

Frequencies and percentages were calculated for categorical variables. Mean and standard deviation (SD) were calculated for continuous variables. Chi-squared test compared between-group differences for categorical variables (i.e., sex, race, geography) and two-tailed unpaired *T*-tests compared between-group differences for continuous variables (i.e., age). Bonferroni correction was applied to adjust for multiple comparisons. Statistical significance was defined as *P* < 0.05.

## Results

The study population included 2,392,855 members of a large national payor, comprised of 1,928,106 adults and 464,749 children. The median duration of continuous plan enrollment was 10 mo across all cohorts. Major outcomes are segmented by age (i.e., adults and children) and neurodevelopmental (i.e., ASD, ADHD, AuDHD) cohorts and presented as follows: prevalence and sociodemographic characteristics; co-occurring diagnoses; healthcare utilization; and healthcare expenditures.

### Prevalence and sociodemographic characteristics

Tables 1, 2 details prevalence and sociodemographic characteristics among the total evaluated population and by neurodevelopmental cohort for adults and children, respectively.

**Table 1:** Prevalence and sociodemographic characteristics of the total adult population and by neurodevelopmental cohort

Characteristic	Total population (*N* = 1,928,106)	Comparison (*n* = 1,848,191)	ASD (*n* = 2,134)	ADHD (*n* = 76,515)	AuDHD (*n* = 1,266)
Prevalence			0.11%	3.97%	0.06%
Age ± SD (years)	40.4 ± 13.4	40.6 ± 13.4	27.5 ± 10.1 ***	34.4 ± 10.8 ***	25.3 ± 8.3 ***
Gender (%)					
Male	48.9	49.0	68.7 ***	46.1 ***	67.8 ***
Female	51.1	51.0	31.2 ***	53.8 ***	32.0***
Unknown	0.1	0.1	0.1	0.1*	0.2*
Race / Ethnicity (%)					
White (Non-Hispanic)	43.8	43.1	52.7 ***	60.4 ***	60.0 ***
Black	6.3	6.4	3.7 ***	3.4 ***	2.8 ***
Hispanic	7.5	7.6	4.7 ***	5.2 ***	3.2 ***
Asian	5.4	5.5	4.1*	2.7 ***	2.1 ***
AI/AN	0.2	0.2	0.2	0.2	0.0
PI	0.1	0.1	0.3	0.1	0.0
Other	3.2	3.2	3.0	3.9 ***	3.5
Missing	33.4	33.8	31.2	24.2***	28.5***
Geography (%)					
Midwest	12.4	12.4	12.6	12.5	14.8
Northeast	24.3	24.3	33.4***	24.9**	29.5***
South	40.7	40.7	31.4***	41.7***	34.2***
West	22.5	22.6	22.6	20.9***	21.5

Abbreviations: ASD, autism spectrum disorder; ADHD, attention-deficient/hyperactivity disorder; AuDHD; co-existing ASD and ADHD; AI, American Indian; NA, Native American; PI, Pacific Islander; SD, standard deviation

*P < 0.05, **P < 0.01, ***P < 0.001, neurodevelopmental vs. comparison cohort

**Table 2:** Prevalence and sociodemographic characteristics of the total pediatric population and by neurodevelopmental cohort

Characteristic	Total population (*N* = 464,749)	Comparison (*n* = 433,736)	ASD (*n* = 5,098)	ADHD (*n* = 23,250)	AuDHD (*n* = 2,665)
Prevalence			1.10%	5.00%	0.57%
Age ± SD (years)	8.6 ± 5.3	8.4 ± 5.3	8.2 ± 4.4 ***	12.1 ± 3.4 ***	11.3 ± 3.5 ***
Gender (%)					
Male	51.0	49.9	75.3 ***	64.6***	77.6 ***
Female	48.9	50.1	24.7 ***	35.4***	22.4 ***
Unknown	0.1	0.0	0.0	0.0	0.0
Race (%)					
White (Non-Hispanic)	39.3	38.7	39.0	49.8***	47.3 ***
Black	4.5	4.5	6.2 ***	3.8***	4.4
Hispanic	5.0	5.1	7.2 ***	4.3***	4.6
Asian	4.4	4.5	6.7 ***	1.4***	4.2
AI/AN	0.2	0.2	0.1	0.2	0.2
PI	0.0	0.1	0	0	0
Other	3.1	3.1	3.3	3.3	3.4
Missing	43.5	43.9	37.4 ***	37.2***	35.9 ***
Geography (%)					
Midwest	13.4	13.3	10.8 ***	14.7 ***	13.5
Northeast	23.3	23.2	26.9 ***	23.1	29.3 ***
South	39.8	39.6	39.0	44.9 ***	38.0
West	23.5	23.9	23.4	17.3 ***	19.2 ***

Abbreviations: ASD, autism spectrum disorder; ADHD, attention-deficient/hyperactivity disorder; AuDHD; co-existing ASD and ADHD; AI, American Indian; NA, Native American; PI, Pacific Islander; SD, standard deviation

*P < 0.05, **P < 0.01, ***P < 0.001, neurodevelopmental vs. comparison cohort

### Adults

Among members ≥ 18 year (*N* = 1,928,106), 4.2% of adults were diagnosed with neurodevelopmental disorders: ADHD (4.0%, *n* = 76,515); ASD (0.1%, *n* = 2,134); or AuDHD (0.1%, *n* = 1,266) (all *P* < 0.001). On average, the neurodevelopmental cohort (*N* = 79,915) was predominately White (60.2%), female (52.9%) young adults (mean age: 34.1 ± 10.9 year [yr]; range: 18.0–69.0 year) residing across South (41.3%), Northeast (25.2%), West (20.9%), and Midwest (12.6%) geographies.

The comparison cohort (95.9%, *n* = 1,848,191) was predominately White (43.1%), female (51.0%) and male (49.0%) adults (mean age: 40.6 ± 13.4 year; range: 18–99 year) residing across South (40.7%), Northeast (24.3%), West (22.6%), and Midwest (12.4%) geographies.

### Children

Among members < 18 year (*N* = 464,749), 6.7% of children were diagnosed with neurodevelopmental disorders: ADHD (5.0%, *n* = 23,250); ASD (1.1%, *n* = 5,098); or AuDHD (0.6%, *n* = 2,665) (all *P* < 0.001). On average, the neurodevelopmental cohort (*N* = 31,013) was predominately White (47.8%), male (67.5%) and female (32.5%) children (mean age: 11.3 ± 3.8 year; range: 0.0–17.0 year) residing across South (43.4%), Northeast (24.2%), West (18.5%), and Midwest (14.0%) geographies.

The comparison cohort (93.3%, *N* = 433,736) was predominately White (38.7%), female (50.1%) and male (49.9%) children (mean age: 8.4 ± 5.3 year; range: 0–17 year) residing across South (39.6%), West (23.9%), Northeast (23.2%), and Midwest (13.3%) geographies.

#### Co-occurring diagnoses and comorbidities

Supplementary Tables 1–6 detail co-occurring diagnoses and comorbidities among members with neurodevelopmental disorders for adults and children.

### Adults

Among adults with neurodevelopmental disorders, there were greater odds (i.e., OR ≥ 2) for certain co-occurring diagnoses that were consistently observed across all three neurodevelopmental cohorts (Supplementary Tables 1–3). A majority of co-occurring diagnoses were BH-related, including: anxiety, avoidant/restrictive food intake disorder, bipolar disorder, depression, eating disorders, gender dysphoria, post-traumatic stress disorder (PTSD), psychiatric disorders related to medical conditions (e.g., mood disorders, conversion disorder, etc.), and personality disorders (e.g., histrionic, obsessive-compulsive, factitious, borderline, paranoid, and other personality disorders), suicidal ideation, and suicide attempt. In addition, adults with ASD and/or ADHD had higher odds (i.e., OR *≥* 2) for co-occurring neurological, autoimmune, and/or gastrointestinal clinical conditions and disorders.

### Children

Among children with neurodevelopmental disorders, there were greater odds (i.e., OR ≥ 2) for certain co-occurring diagnoses that were that were consistently observed across all three neurodevelopmental cohorts (Supplementary Tables 4–6). Similar to adults, many co-occurring diagnoses were BH-related, including alcohol use disorder, anxiety, avoidant/restrictive food intake disorder, bipolar disorder, depression, disruptive childhood disorders, eating disorders, gender dysphoria, PTSD, psychiatric disorders related to medical conditions, schizophrenia, personality disorders, and suicidal ideation. In addition, children with neurodevelopmental disorders had consistently greater odds for non BH-related co-occurring diagnoses such as cerebrovascular disease, epilepsy, chronic thyroid disorders, obesity, and sleep disorders.

#### Healthcare utilization

Table 3 details differences in healthcare utilization among neurodevelopmental (versus comparison) cohorts in adults and children.

**Table 3:** Difference in healthcare utilization in neurodevelopmental (vs. comparison) cohorts

Sub-Category	Mean Difference (95% CI) in Healthcare Utilization (PTPM)^a^					
	ASD		ADHD		AuDHD	
Adults						
ED	**4.3**	(1.0, 7.5)*	**4.3**	(3.7, 4.9)**	**6.6**	(2.3, 10.9)**
PCP	**59.5**	(43.8,75.2)**	**116.4**	(113.7, 119.1)**	**94.8**	(74.3, 115.2)**
Specialist	**42.7**	(15.7, 69.7)**	**91.4**	(86.8, 96.0)**	**77.2**	(42.1, 112.4) **
BH - ED	**105.5**	(100.6, 110.3)**	**141.7**	(140.9, 142.5)**	**210.9**	(204.6, 217.3)**
BH - PCP	**51.0**	(46.8, 55.2)**	**5.4**	(4.7, 6.1)**	**47.8**	(42.3, 53.1)**
BH - specialist	**526.7**	(496.0, 557.4)**	**316.0**	(310.8, 321.2)**	**698.6**	(658.7, 738.6)**
Total	**711.3**	(663.9, 758.8)**	**615.2**	(607.1, 623.3)**	**1024.8**	(963.0, 1086.6)**
Children						
ED	**0.2**	(7.2, 11.2)**	**3.8**	(2.8,4.7)**	**7.0**	(4.2, 9.7)**
PCP	**37.1**	(15.5, 58.6)**	**-13.2**	(-23.4, -3.0)*	**25.4**	(-4.1, 54.9)
Specialist	**112.7**	(99.0, 126.4)**	**53.6**	(47.2, 60.1)**	**138.1**	(119.4, 156.8)**
BH - ED	**137.4**	(134.9, 139.9)**	**78.6**	(77.4, 79.8)**	**191.0**	(187.6, 194.4)**
BH - PCP	**51.5**	(47.2, 55.7)**	**12.6**	(10.6, 14.6)**	**58.1**	(52.3, 64.0)**
BH - specialist	**1162.6**	(1146.0, 1179.2)**	**297.1**	(289.2, 304.9)**	**947.9**	(925.1, 970.6)**
Total	**1205.3**	(1173.9, 1236.7)**	**398.4**	(383.7, 413.2)**	**1184.5**	(1141.6, 1227.4)**

Abbreviations: ASD, autism spectrum disorder; ADHD, attention-deficient/hyperactivity disorder; AuDHD; co-existing ASD and ADHD; BH, behavioral health; ED, emergency department; CI, confidence interval; PCP, primary care provider; PTPM, per thousand per month; *P < 0.05, **P < 0.001 (after Bonferroni correction), neurodevelopmental vs. comparison cohort

^a^Between-group difference calculated as average healthcare utilization for each neurodevelopmental cohort minus comparison cohort; positive values indicate higher healthcare utilization for the neurodevelopmental cohort versus comparison cohort. Note that BH-related ED, PCP, and specialist visits are mutually exclusive. Total healthcare utilization reflects additional sub-categories not included in this analysis

### Adults

Adults with neurodevelopmental disorders had consistently higher rates of healthcare utilization across all neurodevelopmental groups (Table 3). Compared to the comparison cohort, total healthcare utilization was 711.3 (95% CI: 663.9, 758.8), 615.2 (95% CI: 607.1, 623.3), and 1024.8 (95% CI: 963.0, 1086.6) PTPM higher among adults with ASD, ADHD, and AuDHD, respectively (all *P* < 0.001). These trends persisted for BH-related visits, such that total BH-related healthcare utilization was 545.6 (95% CI: (520.9, 570.4), 389.5 (95% CI: 385.3, 393.7), and 791.2 (95% CI: 759.0, 823.5) PTPM higher among adults with ASD, ADHD, and AuDHD, respectively (all *P* < 0.001). In addition, differences in healthcare utilization were generally consistent across all sub-categories with the highest rates observed for BH-related specialist visits.

### Children

Children with neurodevelopmental disorders had consistently higher rates of healthcare utilization across all neurodevelopmental groups (Table 3). Compared to the comparison cohort, total healthcare utilization was 1205.3 (95% CI: 1173.9, 1236.7), 398.4 (95% CI: 383.7, 413.2), and 1184.5 (95% CI: 1141.6, 1227.4) PTPM higher among children with ASD, ADHD, and AuDHD, respectively (all *P* < 0.001). These trends persisted for BH-related visits, such that total BH-related healthcare utilization was 827.4 (95% CI: 814.6, 840.1), 329.9 (95% CI: 323.9, 336.0), and 866.6 (95% CI: 849.1, 884.1) PTPM higher among children with ASD, ADHD, and AuDHD, respectively (all *P* < 0.001). Differences in healthcare utilization were generally consistent across all sub-categories with the highest rates observed for BH-related specialist visits (both *P* < 0.001). Of note, children with ADHD had − 12.2 PTPM fewer observed PCP visits compared to the comparison cohort (*P* < 0.05).

#### Healthcare expenditures

Table 4 details differences in healthcare expenditures among neurodevelopmental (versus comparison) cohorts in adults and children.

**Table 4:** Difference in healthcare expenditures in neurodevelopmental (vs. comparison) cohorts

Sub-Category	Mean Difference (95% CI) in Healthcare Expenditures (PMPM)^a^					
	ASD		ADHD		AuDHD	
Adults						
ED	**6.9**	(0, 14.9)	**9.9**	(8.6,11.3)**	**10.2**	(0, 20.6)
PCP	**8.9**	(7.2, 10.8)**	**15.6**	(15.4, 16.0)**	**13.3**	(10.9, 15.6)**
Specialist	**11.6**	(2.8, 20.3)*	**16.5**	(15.0, 18.0)**	**11.1**	(0, 22.5)
BH - ED	**13.9**	(13.3, 14.5)**	**16.6**	(16.5, 16.7)**	**26.3**	(25.6, 27.1)**
BH - PCP	**4.1**	(3.4, 4.8)**	**0.8**	(0.7, 0.9)**	**8.8**	(7.8, 9.7)**
BH - Specialist	**65.0**	(61.3, 68.7)**	**37.6**	(37.0, 38.2)**	**85.1**	(80.3, 90.0)**
Total	**232.2**	(188.9, 275.6)**	**140.3**	(132.9, 147.6)**	**292.1**	(235.7, 348.4)**
Children						
ED	**14.2**	(10.1, 18.2)**	**9.0**	(7.1, 10.9)**	**10.2**	(4.7, 2.9)**
PCP	**6.8**	(5.1, 8.6)	**4.2**	(3.5, 5.1)**	**9.6**	(7.2, 12.0)**
Specialist	**30.3**	(26.3, 34.3)**	**13.4**	(11.5, 15.3)**	**38.9**	(33.4, 44.4)**
BH - ED	**19.9**	(19.6, 20.2)**	**10.5**	(10.3, 10.6)**	**25.8**	(25.3, 26.3)**
BH - PCP	**14.4**	(13.3, 15.5)**	**1.5**	(1.1, 2.1)**	**8.3**	(6.8, 9.8)**
BH - Specialist	**146.7**	(144.7, 148.8)**	**36.1**	(35.1, 37.0)**	**117.7**	(115.0, 120.5)**
Total	**845.4**	(818.6, 872.2)**	**50.8**	(38.1, 63.4)**	**574.5**	(537.8, 611.2)**

Abbreviations: ASD, autism spectrum disorder; ADHD, attention-deficient/hyperactivity disorder; AuDHD; co-existing ASD and ADHD; BH, behavioral health; ED, emergency department; CI, confidence interval; PCP, primary care provider; PMPM, per member per month; *P < 0.05, **P < 0.001 (after Bonferroni correction), neurodevelopmental vs. comparison cohort

^a^Between-group difference calculated as average healthcare expenditures for each neurodevelopmental cohort minus comparison cohort; positive values indicate higher healthcare spend for the neurodevelopmental cohort versus comparison cohort. Note that BH-related ED, PCP, and specialist visits are mutually exclusive. Total healthcare expenditures reflect additional sub-categories not included in this analysis

### Adults

Adults with neurodevelopmental disorders had consistently higher rates of healthcare expenditures across all neurodevelopmental groups (Table 4); patterns that were parallel to observed differences in healthcare utilization (Table 3). Compared to the comparison cohort, total healthcare spend was $232.20 (95% CI: 188.9, 275.6), $140.30 (95% CI: 132.9, 147.6), and $292.1 PMPM (95% CI: 235.7, 348.4) higher among individuals with ASD, ADHD, and AuDHD, respectively, than the comparison cohort (all *P* < 0.001). These trends persisted for BH-related visits, such that total BH-related healthcare expenditures were $82.8 (95% CI: 79.1, 86.4), $59.3 (95% CI: 58.7, 60.0), and $123.9 (95% CI: 119.2, 128.7) PMPM higher among adults with ASD, ADHD, and AuDHD, respectively (all *P* < 0.001). In addition, differences in healthcare expenditures were predominately driven by increased spend for BH-specialist visits. Notably, there were no between-group difference in expenditures for ED visits in adults with AuDHD or ASD (both *P ≥* 0.14).

### Children

Children with neurodevelopmental disorders had consistently higher rates of healthcare expenditures across all neurodevelopmental groups (Table 4); patterns that were parallel to observed differences in healthcare utilization (Table 3). Compared to the comparison cohort, total healthcare spend was $845.40 (95% CI: 818.6, 872.2), $50.80 (95% CI: 38.1, 63.4), and $574.50 PMPM (95% CI: 537.8, 611.2) higher among children with ASD, ADHD, and AuDHD, respectively (all *P* < 0.001). These trends persisted for BH-related visits, such that total BH-related healthcare expenditures were $133.70 (95% CI: 131.7, 135.6), $52.00 (95% CI: 51.1, 52.9), and $140.80 (95% CI: 138.1, 143.4) PMPM higher among children with ASD, ADHD, and AuDHD, respectively (all *P* < 0.001). In addition, differences in healthcare expenditures were predominately driven by increased spend for BH-related specialist visits. Note that there was no between-group difference in expenditures for PCP visits in children with ASD (*P* = 0.93).

## Discussion

### Principal findings

This retrospective cohort analysis of 2,392,855 C-FI members of a large national payor sought to evaluate the prevalence, sociodemographic composition, and top co-occurring diagnoses among adult and pediatric members of a large national health plan with a diagnosis of ASD and/or ADHD. As a secondary aim, differences in healthcare utilization and related expenditures between members with and without these neurodevelopmental disorders were explored. Major findings were that 4.2% of adults and 6.7% of children were diagnosed with ASD and/or ADHD and these members exhibited higher odds of co-occurring conditions and greater healthcare utilization and expenditures compared to members without these neurodevelopmental disorders. Notably, members with ASD and/or ADHD consistently showed increased use of BH-related services, which accounted for a majority of increased expenditures observed in this population.

### Interpretation of principal findings

The neurodevelopmental prevalence rates for ASD and/or ADHD observed in this study are consistent with prior research [2, 4–6]; however, slight differences may reflect variation in in diagnostic recognition, reporting source, and data collection methodology. For example, ADHD prevalence among children is estimated to be ~ 11% based on parent-reported survey data from 4 to 8 year old children across 15 surveillance states [5], whereas the present study identified a prevalence of ~ 6% based on real-world administrative claims. Notably, this study advances the literature by characterizing the prevalence of ASD and/or ADHD in adults, a population often underrepresented in neurodevelopmental research [4]. The identification of 4.2% of adults (*n* = 79,915) with ASD and/or ADHD underscores the importance of addressing the needs of neurodevelopmental disorders across the lifespan [27].

The number of individuals diagnosed with AuDHD in our study population were comparable to prevalence rates previously reported, whereby 52.3% of children and 59.3% of adults with ASD had a co-existing diagnosis of ADHD. These results are slightly higher than those previously reported in the literature; for example, in a recent claims-based analysis involving 6719 adults and children, 44.2–50.1% of individuals with ASD had co-occurring ADHD [9]. In a study exclusively conducted in children (*N* = 2,495) with ADHD, 13% had co-occurring ASD [8], which is similar to our finding that 11.5% of children with ADHD had a co-existing diagnosis of ASD. Interestingly, in the present study, 1.7% of adults with ADHD had a co-existing diagnosis of ASD, which is markedly lower than 16% previously reported by Pehlivanidis et al., which characterized co-occurring diagnoses among adults in Greece with newly diagnosed neurodevelopmental disorders who underwent thorough clinical evaluations using standardized diagnostic tools [31]. Reasons for this discordance are likely owing to methodological differences (i.e., standard healthcare practices versus a clinical study setting designed to detect co-occurring diagnoses). Nevertheless, the lower prevalence of ASD in adults with ADHD may signal potential underdiagnosis and warrants additional exploration.

The sociodemographic composition of members with ASD and/or ADHD revealed notable differences in geographic distribution and gender. Consistent with existing literature [3, 32, 33], males were overrepresented in the pediatric population across all neurodevelopmental groups (Tables 2 and 64.6 to 77.6%). Interestingly, females comprised 53.8% of the adult ADHD cohort, which may reflect increasing recognition and diagnosis of ADHD in females; a group historically underdiagnosed [34]. The concentration of neurodevelopmental disorder diagnoses in southern and northeastern regions may reflect regional differences in healthcare access, diagnostic practices, or cultural attitudes towards neurodevelopmental disorders and requires additional research to better understand these geographic disparities.

Adults and children with ASD, ADHD, or AuDHD appear to be at significant risk for a wide array of comorbid conditions, spanning behavioral, psychiatric, and clinical domains. The exceptionally higher OR values in adults and children with AuDHD suggests that co-occurring ASD and ADHD may synergistically increase odds of additional health-related challenges. These findings reinforce the importance of a comprehensive, multidisciplinary approach to treatment that includes medical, behavioral, and support services tailored to the complex needs of the individual. In some instances, common co-occurring conditions among individuals with neurodevelopmental disorders may be amenable to shared interventions, particularly where underlying mechanisms overlap or where one conditions exacerbates another. Integrating evidence-based interventions with common therapeutic goals such as Cognitive Behavioral Therapy, medication management, or supportive lifestyle changes, can potentially address multiple overlapping conditions [10]. Such multidisciplinary approaches have strong potential to improve patient-related outcomes (i.e., experience, quality of life, and adherence), reduce healthcare encounters and related expenditures; and ultimately improve health and healthcare outcomes.

As hypothesized, healthcare utilization and expenditures were higher in members with neurodevelopmental disorders compared to the comparison cohort. Additionally, members with ASD (with or without co-occurring ADHD) demonstrated higher overall expenditures compared to those with isolated ADHD. These differences may reflect more complex care needs, higher rates of comorbid conditions, greater reliance on specialist services, and/or increased intensity of care coordination. Differences in healthcare utilization and expenditures were predominately driven by BH-related services for both age cohorts (i.e., children and adults) and across all neurodevelopmental groups (i.e., ASD, ADHD, and AuDHD). For example, children with ASD had healthcare expenditures nearly $845 PMPM higher (than children without ASD) with BH-related services representing a majority of this difference. Similarly, adults with ASD and/or ADHD incurred up to $292 PMPM more in total healthcare costs; predominately driven by BH-related expenditures. These findings are similar in direction, but lower in magnitude to those reported in the literature. For example, a recent systematic review and meta-analysis involving 32 studies reported that children with ADHD had annual healthcare costs $4,167 higher than children without ADHD [35].

The high utilization of BH-related services raises important questions about the appropriateness and scope of care. For example, preliminary *post hoc* analysis revealed a large proportion of expenditures (i.e., ~ 60%) for children with ASD and/or ADHD were attributed to ABA therapy; an evidence-based intervention with strong focus on short-term behavioral outcomes, but by definition does not address broader emotional and mental health of this population, such as anxiety and depression [36].This data suggests the need for additional research into the best ways to address common comorbidities, inclusive of physical emotional and mental health, in a holistic and comprehensive way.

### Real-world implications

The results of this evaluation have several implications for healthcare providers, payors, researchers, policymakers, and employers. These findings raise important questions about whether the higher utilization and expenditures observed reflect unmet needs or if they are an artifact of the higher volume of encounters required to provide adequate care. This ambiguity emphasizes the need for further research to examine whether current healthcare practices and interventions sufficiently address the complex needs of individuals with neurodevelopmental disorders. Additional study is also needed to evaluate the unique lived experience of these members in the healthcare system.

From an economic perspective, the increased healthcare costs associated with neurodevelopmental disorders are significant. Put into context, these differences translate to approximately $223.5 million (USD) in *additional* healthcare costs for the included sample of ~ 111k members with ASD and/or ADHD annually. It is important to note that this analysis does not account for out-of-pocket expenditures or indirect costs, such as productivity losses, thus the full economic impact of neurodevelopmental disorders on individuals, employers, and the healthcare system is likely underestimated. Understanding and addressing potentially unmet needs through personalized interventions and care coordination has strong potential to improve health outcomes and reduce unnecessary medical spend and warrants further research [37, 38]. Additionally, there is growing interest among employers to better accommodate employees (and their families) with neurodevelopmental disorders as part of broader recruitment, retention, and diversity, equity, inclusion, and belonging initiatives [23, 24]. Employers have the potential to play a critical role in reducing healthcare disparities by offering comprehensive benefits, support services, and workplace wellness and employee assistance programs.

Adding to the relatively nascent and variable body of literature [13, 14, 39, 40], this research further demonstrates that individuals with ASD and/or ADHD have disproportionally higher burden of mental health issues, including anxiety, depression, PTSD, and suicidal ideation or attempts. These findings suggest a strong need for more focus on emotional and psychological outcomes in both research and clinical practice [41]. Higher rates of eating disorders, sleep disorders, and gender dysphoria in individuals with neurodevelopmental disorders highlight the importance of neurodiversity-informed clinical practices for these disorders [42]. Treatments for conditions such as avoidant/restrictive food intake disorder or sleep disturbances should accommodate sensory sensitivities common in these populations [43]. Finally, access to neurodiversity informed medical care is a critical concern, as individuals with ASD more likely to experience chronic physical health conditions, higher morbidity, and premature mortality compared to the general population [15, 44, 45]. Enhanced provider education at a national level on the complexities of neurodevelopmental disorders could potentially improve diagnostic and treatment approaches regardless of geographic location. Addressing communication, executive functioning, and sensory needs through standardized training and education initiatives can further diminish barriers to equitable care [22].

### Limitations

There are few noteworthy limitations to this study. This study infers differences in healthcare utilization and expenditures owing to the presence of one or more neurodevelopmental diagnoses. Although these findings were statistically significant, this study was not designed to explore a definitive causal relationship. The large sample size enhances statistical power and generalizability of the findings, but can magnify small differences that are statistically, but not clinically significant. These data do not include detailed information on pharmacy-related expenditures, which limits the ability to assess the financial impact of medication use on study outcomes. The lack of data on subtypes of ASD and/or ADHD limits the ability to explore differences in healthcare utilization and costs based on the severity of neurodevelopmental disorders. This study does not differentiate between employed and non-employed adults with ASD and/or ADHD, which limits the ability to draw conclusions about how workforce engagement may affect healthcare utilization and outcomes. Gender is self-reported during plan enrollment, therefore, this study is limited in its ability to differentiate between gender, sex assigned at birth, and legal sex. Additional research is required to fully explore the multiple dimensions and variations of sex (as a biological variable) and gender (as a social and cultural variable) that independently and interactively influence the full spectrum of health and healthcare. Lastly, claims data may not capture all relevant information, such as self-reported impacts on health and healthcare. In addition, claims-based, point prevalence estimates may not fully capture individuals with delayed, underdiagnosed, or undocumented conditions. However, this approach allows for the identification of active cases within a defined time frame and enables cross-sectional comparisons of clinical and healthcare utilization outcomes across population subgroups. Relatedly, a small proportion of members had partial-year enrollment with the following percentile distribution: 10th: 2 mo; 25th: 5 mo; 50th: 10 mo; 75th: 12 mo; 90th: 12 mo). This variation may have confounded group comparisons. However, utilization and expenditures were annualized to account for differences in enrollment duration. Notably, the majority of members had full-year enrollment (i.e., 12 mo), and similar average and percentile distributions were observed across all cohorts: ADHD (10th: 3 mo; 25th: 6 mo; 50th: 12 mo; 75th: 12 mo; 90th: 12 mo), ASD and AuDHD (10th: 4 mo; 25th: 7 mo; 50th: 12 mo; 75th: 12 mo; 90th: 12 mo), and comparison (10th: 2 mo; 25th: 5 mo; 50th: 10 mo; 75th: 12 mo; 90th: 12 mo).

Despite the noted limitations, this study possesses several strengths. This evaluation represents a rigorously designed retrospective, cohort study in a large, geographically diverse sample size of nearly 2.4 M individuals. The integration of real-world data contributes to a more holistic and ecologically valid understanding of the clinical outcomes, member experience, healthcare utilization, and cost dynamics among individuals with neurodevelopmental diagnoses. To the best of our knowledge, there are limited studies to examine these outcomes in both adults and children. Access to member claims data enables quantification of healthcare spend without relying on simulated models or estimates. Finally, the study cohort is a representative population for whom future health plan and/or employer sponsored initiatives would be used by (i.e., C-FI members of a large national health plan and their families). As our understanding of this population continues to evolve, this study provides a benchmark for expected differences and opportunities to better support individuals with ASD and/or ADHD.

### Future research

This study provided a naturalistic, observational evaluation of real-world prevalence, co-occurring diagnoses, and healthcare utilization and expenditures among individuals with ASD, ADHD, or both. The primary objective was to describe patterns of healthcare use within naturally occurring populations. Accordingly, propensity score matching or other statistical adjustment procedures were intentionally not applied, as such methods would alter the underlying population structure and reduce the generalizability of findings by excluding members with valid but unmatched characteristics. The resulting estimates reflect actual differences observed in a real-world setting.

Building on these foundational insights, immediate next steps include follow-up studies aimed at better characterizing the complex relationships between healthcare service delivery (i.e., volume, type, etc.) and outcomes segmented by age, diagnostic group, and other social determinants of health factors. For example, observed differences in racial and ethnic composition across cohorts may reflect a range of intersecting factors, including disparities in diagnostic access, cultural or systemic bias in healthcare settings, variation in help-seeking behaviors, and differential access to specialist services. In addition, biological and sociodemographic variables - such as genetic predisposition(s), age, sex/gender, race/ethnicity, and geographic location - may influence not only the development of neurodevelopmental disorders and co-occurring diagnoses, but also the likelihood of receiving a diagnosis and subsequent healthcare utilization. Future research employing multivariable modeling or matched cohort designs represent a logical progression to examine these interrelated factors and advance our understanding of how they influence diagnostic pathways and health-and healthcare-related outcomes across diverse populations.

Further analysis of coexisting conditions offers significant potential for advancing the development of archetypes or phenotypes within neurodevelopmental disorders like ASD and/or ADHD. These advanced models can be used to better screen, identify, and support individuals with these conditions. Identifying clusters of frequently co-occurring diagnoses and comorbidities, would enable researchers and clinicians to categorize individuals into more specific subgroups and ultimately enable person-centered and value-based care. Such models could potentially improve diagnostic accuracy and earlier identification of high-risk individuals. Additionally, these models could inform the development of integrated care plans that address multiple comorbidities simultaneously.

Understanding the impact of personalized interventions on healthcare utilization and outcomes for individuals with ASD and/or ADHD would be of great public health interest. For example, outcome-based studies that examine the long-term effects of integrated care models (that address both behavioral and medical comorbidities) could potentially inform healthcare policy and practice.

Finally, given the growing focus on neurodevelopmental disorders among employers, research into the effectiveness of workplace-based interventions and support for employees and their families is sorely needed. Collectively, these findings could ultimately guide the development of policies aimed at improving healthcare outcomes, reducing disparities, and enhancing employee retention and satisfaction. Additional exploration into the differences between subscriber and dependents and working and non-working adults, particularly in the context of employer-sponsored health plans, is an important distinction for understanding the extent to which employment status impacts access to care and downstream outcomes.

## Conclusions

Leveraging real-world data from a large national payor, 4.2% of adults and 6.7% of children were diagnosed with ASD and/or ADHD. Individuals with these neurodevelopmental disorders have markedly higher observed healthcare utilization and expenditures compared to those without. Lastly, individuals with these ASD and/or ADHD have higher odds of specific comorbidities and diagnoses that frequently co-occur, requiring an interdisciplinary approach.

## Supplementary Information

Below is the link to the electronic supplementary material.

Supplementary Material 1

## Data Availability

The data sets generated during and/or analyzed during the current study are not publicly available due to commercial data use agreements.

## Abbreviations

ADHD: Attention-deficit/hyperactivity disorder
AuDHD: Co-existing autism spectrum disorder and attention-deficit/hyperactivity disorder
ASD: Autism spectrum disorder
BH: Behavioral health
C-FI: Commercial fully insured
CI: Confidence interval
CPT-4: Current Procedure Terminology 4th revision
ED: Emergency department
HPD: Health Profile Database
ICD-10: 10th Revision of the International Classification of Diseases
OR: Odds ratio
PCP: Primary care provider
PMPM: Per member per month
PTPM: Per thousand per month
PTSD: Post-traumatic stress disorder
SD: Standard deviation
US: United States
USD: US dollar
