# Supplementary material for: Real-world evaluation of prevalence, cohort characteristics, and healthcare utilization and expenditures among adults and children with autism spectrum disorder, attention-deficit hyperactivity disorder, or both
Source: BMC Health Serv Res. 2025 Aug 9;25:1048. doi: 10.1186/s12913-025-13296-2 (PMC12335152; doi:10.1186/s12913-025-13296-2)
Supplement: Supplementary file 1 — Supplementary Material 1 [file 12913_2025_13296_MOESM1_ESM.docx]

# Supplementary Tables 1‒6. Odds Ratios and Prevalence of Co-Existing Diagnoses Among Adults and Children with Neurodevelopmental Disorders Compared to the Comparison Cohort

**Supplementary Table 1. Odds Ratios with 95% Confidence Interval for Co-Existing Diagnoses Among Adults with ASD**

|  |  |  | **Cohort Prevalence (%)** | |
| --- | --- | --- | --- | --- |
| **Co-Existing Diagnoses** | **OR^a^** | **95 CI% (lower, upper)** | **ASD** | **Comparison** |
| Disruptive childhood disorders | 76.3 | (56.8, 102.5) | 2.3 | 0.0 |
| Down’s syndrome | 73.1 | (43.8, 121.9) | 0.7 | 0.0 |
| Avoidant/restrictive food intake disorder | 30.3 | (9.6, 95.7) | 0.1 | 0.0 |
| Epilepsy | 21.3 | (18.5, 24.5) | 10.1 | 0.5 |
| Schizophrenia | 20.9 | (16.5, 26.5) | 3.4 | 0.2 |
| Suicidal ideation | 16.7 | (12.5, 22.3) | 2.2 | 0.1 |
| Gender dysphoria | 16.7 | (12.3, 22.7) | 2.0 | 0.1 |
| Specific personality disorders | 14.2 | (10.5, 19.1) | 2.1 | 0.2 |
| Suicide attempt | 13.2 | (5.9, 29.6) | 0.3 | 0.0 |
| Bipolar disorder | 8.8 | (7.5, 10.3) | 7.6 | 0.9 |
| Cystic fibrosis | 8.8 | (2.2, 35.3) | 0.1 | 0.0 |
| Eating disorders | 5.7 | (4.1, 7.9) | 1.7 | 0.3 |
| Psychiatric disorders related to medical conditions | 5.5 | (4.5, 6.8) | 4.5 | 0.8 |
| Anxiety | 5.3 | (4.9, 5.8) | 48.5 | 15.0 |
| Low vision and blindness | 4.6 | (2.7, 7.8) | 0.7 | 0.1 |
| Depression | 4.5 | (4.1, 4.9) | 31.7 | 9.3 |
| Congenital heart disease | 4.5 | (3.2, 6.3) | 1.5 | 0.3 |
| Post-traumatic stress disorder | 4.1 | (3.2, 5.3) | 2.7 | 0.7 |
| Brain cancer | 4.0 | (1.5, 10.7) | 0.2 | 0.0 |
| Non-Alzheimer’s dementia | 3.1 | (1.7, 5.8) | 0.5 | 0.2 |
| Inflammatory bowel disease | 2.9 | (2.2, 4.0) | 2.0 | 0.7 |
| Fibromyalgia | 2.9 | (2.0, 4.2) | 1.3 | 0.5 |
| Non-infectious hepatitis | 2.6 | (1.5, 4.4) | 0.6 | 0.2 |
| Otitis media | 2.1 | (1.7, 2.5) | 5.7 | 2.8 |
| Asthma | 2.0 | (1.8, 2.4) | 9.6 | 4.9 |

Abbreviations: ASD, autism spectrum disorder; CI, confidence interval; OR, odds ratio

**^a^** Includes all OR values >2.0 (ranked; all *P*=0.000)

# Supplementary Table 2. Odds Ratios with 95% Confidence Interval for Co-Existing Diagnoses Among Adults with ADHD

|  |  |  | **Cohort Prevalence (%)** | |
| --- | --- | --- | --- | --- |
| **Co-Existing Diagnoses** | **OR^a^** | **95 CI% (lower, upper)** | **ADHD** | **Comparison** |
| Disruptive childhood disorders | 15.5 | (13.6, 17.7) | 0.5% | 0.0% |
| Avoidant/restrictive food intake disorder | 7.9 | (5.1, 12.1) | 0.0% | 0.0% |
| Specific personality disorders | 7.4 | (6.9, 8.0) | 1.1% | 0.2% |
| Eating disorders | 7.1 | (6.7, 7.5) | 2.1% | 0.3% |
| Bipolar disorder | 6.9 | (6.7, 7.2) | 6.1% | 0.9% |
| Anxiety | 5.7 | (5.7, 5.8) | 50.3% | 15.0% |
| Depression | 5.7 | (5.6, 5.8) | 36.9% | 9.3% |
| Suicide attempt | 5.4 | (4.3, 6.8) | 0.1% | 0.0% |
| Gender dysphoria | 5.1 | (4.6, 5.6) | 0.6% | 0.1% |
| Suicidal ideation | 4.8 | (4.4, 5.3) | 0.7% | 0.1% |
| Post-traumatic stress disorder | 4.6 | (4.4, 4.9) | 3.1% | 0.7% |
| Connective tissue disorder | 4.1 | (3.3, 5.1) | 0.1% | 0.0% |
| Substance related disorders | 3.9 | (3.8, 4.0) | 5.3% | 1.4% |
| Schizophrenia | 3.6 | (3.2, 3.9) | 0.6% | 0.2% |
| Hereditary angioedema | 3.0 | (1.9, 4.8) | 0.0% | 0.0% |
| Psychiatric disorders related to medical conditions | 3.0 | (2.8, 3.1) | 2.5% | 0.8% |
| Alcohol use disorder | 2.9 | (2.8, 3.0) | 4.2% | 1.5% |
| Non-Alzheimer’s dementia | 2.8 | (2.5, 3.2) | 0.4% | 0.2% |
| Chronic fatigue syndrome | 2.5 | (2.3, 2.6) | 2.1% | 0.8% |
| HIV/AIDS | 2.2 | (2.1, 2.4) | 1.6% | 0.7% |
| Postpartum behavioral health disorder | 2.2 | (2.0, 2.4) | 0.7% | 0.3% |
| Fibromyalgia | 2.2 | (2.0, 2.4) | 1.0% | 0.5% |
| Migraine | 2.0 | (2.0, 2.1) | 8.4% | 4.3% |

Abbreviations: ADHD, attention-deficit/hyperactivity disorder; AIDS, acquired immune deficiency syndrome; CI, confidence interval;

HIV, human immunodeficiency virus; OR, odds ratio

**^a^** Includes all OR values >2.0 (ranked; all *P*=0.000)

# Supplementary Table 3. Odds Ratios with 95% Confidence Interval for Co-Existing Diagnoses Among Adults with AuDHD

|  |  |  | **Cohort Prevalence (%)** | |
| --- | --- | --- | --- | --- |
| **Co-Existing Diagnoses** | **OR^a^** | **95 CI% (lower, upper)** | **AuDHD** | **Comparison** |
| Disruptive childhood disorders | 251.5 | (200.1, 316.1) | 7.2% | 0.0% |
| Avoidant/restrictive food intake disorder | 68.1 | (25.0, 185.9) | 0.3% | 0.0% |
| Down’s syndrome | 38.4 | (15.8, 93.4) | 0.4% | 0.0% |
| Cystic fibrosis | 37.0 | (15.2, 90.0) | 0.4% | 0.0% |
| Suicide attempt | 33.6 | (17.3, 65.2) | 0.7% | 0.0% |
| Gender dysphoria | 28.5 | (20.9, 38.9) | 3.3% | 0.1% |
| Schizophrenia | 28.3 | (21.7, 37.0) | 4.6% | 0.2% |
| Specific personality disorders | 28.2 | (21.3, 37.3) | 4.1% | 0.2% |
| Suicidal ideation | 24.9 | (18.3, 34.0) | 3.3% | 0.1% |
| Connective tissue disorder | 19.1 | (9.0, 40.3) | 0.6% | 0.0% |
| Bipolar disorder | 16.1 | (13.7, 19.0) | 13.1% | 0.9% |
| Anxiety | 12.2 | (10.8, 13.7) | 68.2% | 15.0% |
| Eating disorders | 10.5 | (7.6, 14.4) | 3.1% | 0.3% |
| Epilepsy | 10.2 | (8.0, 13.1) | 5.1% | 0.5% |
| Post-traumatic stress disorder | 9.7 | (7.7, 12.2) | 6.2% | 0.7% |
| Depression | 9.3 | (8.3, 10.4) | 48.9% | 9.3% |
| Psychiatric disorders related to medical conditions | 8.1 | (6.5, 10.1) | 6.4% | 0.8% |
| Non-Alzheimer’s dementia | 5.3 | (2.8, 9.8) | 0.8% | 0.2% |
| Brain cancer | 5.1 | (1.6, 15.8) | 0.2% | 0.0% |
| Congenital heart disease | 4.3 | (2.8, 6.8) | 1.5% | 0.3% |
| Substance related disorders | 4.2 | (3.3, 5.3) | 5.6% | 1.4% |
| Alcohol use disorder | 3.1 | (2.4, 4.1) | 4.5% | 1.5% |
| Sleep disorders | 2.9 | (2.4, 3.5) | 11.1% | 4.1% |
| Asthma | 2.7 | (2.3, 3.2) | 12.2% | 4.9% |
| Fibromyalgia | 2.4 | (1.4, 4.1) | 1.1% | 0.5% |
| Otitis media | 2.4 | (1.9, 3.0) | 6.6% | 2.8% |

| Allergic rhinitis | 2.4 | (2.1, 2.8) | 18.8% | 8.8% |
| --- | --- | --- | --- | --- |

Abbreviations: AuDHD, co-existing autism spectrum disorder and attention-deficit/hyperactivity disorder; CI, confidence interval; OR, odds ratio

**^a^** Includes all OR values >2.0 (ranked; all *P*=0.000)

# Supplementary Table 4. Ratios with 95% Confidence Interval for Co-Existing Diagnoses Among Children with ASD

|  |  |  | **Cohort Prevalence (%)** | |
| --- | --- | --- | --- | --- |
| **Co-Existing Diagnoses** | **OR^a^** | **95 CI% (lower, upper)** | **ASD** | **Comparison** |
| Non-Alzheimer’s dementia | 23.0 | (13.5, 39.4) | 0.3% | 0.0% |
| Cystic fibrosis | 21.1 | (13.2, 33.7) | 0.4% | 0.0% |
| Avoidant/restrictive food intake disorder | 12.6 | (8.1, 19.8) | 0.4% | 0.0% |
| Epilepsy | 12.3 | (10.8, 14.1) | 5.0% | 0.4% |
| Down's syndrome | 9.7 | (7.0, 13.6) | 0.7% | 0.1% |
| Disruptive childhood disorders | 8.5 | (7.4, 9.9) | 4.1% | 0.5% |
| Schizophrenia | 7.4 | (4.1, 13.4) | 0.2% | 0.0% |
| Maternal history of low birth weight or preterm birth | 6.5 | (1.6, 27.6) | 0.0% | 0.0% |
| Alcohol use disorder | 6.5 | (4.0, 10.7) | 0.3% | 0.1% |
| Obstructive sleep apnea | 6.0 | (5.0, 7.2) | 2.4% | 0.4% |
| Sleep disorders | 5.7 | (4.9, 6.7) | 3.7% | 0.7% |
| Eating disorders | 5.7 | (4.5, 7.2) | 1.5% | 0.3% |
| Hodgkin’s disease/lymphoma | 5.4 | (2.2, 13.3) | 0.1% | 0.0% |
| Specific personality disorders | 5.0 | (2.7, 9.3) | 0.2% | 0.0% |
| Leukemia/myeloma | 4.9 | (2.5, 9.6) | 0.2% | 0.0% |
| HIV/AIDS | 4.9 | (1.5, 15.7) | 0.1% | 0.0% |
| Bipolar disorder | 4.6 | (3.1, 7.0) | 0.5% | 0.1% |
| Gender dysphoria | 4.3 | (2.7, 7.1) | 0.3% | 0.1% |
| Psychiatric disorders related to medical conditions | 4.1 | (3.2, 5.3) | 1.3% | 0.3% |
| Cataract | 3.8 | (2.0, 7.4) | 0.2% | 0.0% |
| Ischemic heart disease | 3.7 | (1.4, 10.1) | 0.1% | 0.0% |
| Brain cancer | 3.5 | (1.4, 8.6) | 0.1% | 0.0% |
| Chronic obstructive pulmonary disease | 3.5 | (1.9, 6.6) | 0.2% | 0.1% |
| Low vision and blindness | 3.5 | (2.7, 4.6) | 1.1% | 0.3% |
| Other cancer | 3.4 | (1.7, 6.9) | 0.2% | 0.0% |
| Anxiety | 3.2 | (3.0, 3.5) | 13.7% | 4.7% |

| Congestive heart failure | 3.2 | (1.7, 6.0) | 0.2% | 0.1% |
| --- | --- | --- | --- | --- |
| Chronic renal failure | 3.2 | (1.5, 6.8) | 0.1% | 0.0% |
| Suicidal ideation | 2.6 | (1.8, 3.8) | 0.5% | 0.2% |
| Chronic thyroid disorders | 2.5 | (1.9, 3.1) | 1.4% | 0.6% |
| Obesity | 2.4 | (2.2, 2.6) | 10.7% | 4.8% |
| Cerebrovascular disease | 2.3 | (1.1, 4.7) | 0.2% | 0.1% |
| Depression | 2.2 | (1.9, 2.5) | 4.9% | 2.3% |
| Periodontal disease | 2.2 | (1.4, 3.5) | 0.3% | 0.2% |
| Congenital heart disease | 2.1 | (1.7, 2.5) | 2.2% | 1.1% |
| Post-traumatic stress disorder | 2.0 | (1.2, 3.4) | 0.3% | 0.2% |

Abbreviations: ASD, autism spectrum disorder; AIDS, acquired immune deficiency syndrome; CI, confidence interval; HIV, human immunodeficiency virus; OR, odds ratio

**^a^** Includes all OR values >2.0 (ranked; all *P*=0.000)

# Supplementary Table 5. Ratios with 95% Confidence Interval for Co-Existing Diagnoses Among Children with ADHD

|  |  |  | **Cohort Prevalence (%)** | |
| --- | --- | --- | --- | --- |
| **Co-Existing Diagnoses** | **OR^a^** | **95 CI% (lower, upper)** | **ADHD** | **Comparison** |
| Disruptive childhood disorders | 20.4 | (19.2, 21.7) | 9.2% | 0.5% |
| Bipolar disorder | 11.8 | (10.1, 13.7) | 1.2% | 0.1% |
| Schizophrenia | 10.8 | (8.2, 14.3) | 0.3% | 0.0% |
| Specific personality disorders | 10.0 | (7.8, 12.7) | 0.4% | 0.0% |
| Non-Alzheimer’s dementia | 9.2 | (6.0, 14.1) | 0.1% | 0.0% |
| Anxiety | 9.2 | (8.9, 9.4) | 31.2% | 4.7% |
| Suicidal ideation | 8.5 | (7.6, 9.6) | 1.7% | 0.2% |
| Alcohol use disorder | 8.2 | (6.4, 10.4) | 0.4% | 0.1% |
| Maternal history of low birth weight or preterm birth | 7.9 | (3.9, 16.0) | 0.0% | 0.0% |
| Depression | 7.8 | (7.5, 8.2) | 15.5% | 2.3% |
| Post-traumatic stress disorder | 7.6 | (6.6, 8.7) | 1.2% | 0.2% |
| Suicide attempt | 7.5 | (5.4, 10.4) | 0.2% | 0.0% |
| Brain cancer | 6.7 | (2.4, 18.5) | 0.0% | 0.0% |
| Substance related disorders | 6.5 | (5.7, 7.4) | 1.2% | 0.2% |
| Gender dysphoria | 5.7 | (4.6, 7.1) | 0.4% | 0.1% |
| Psychiatric disorders related to medical conditions | 4.8 | (4.2, 5.4) | 1.5% | 0.3% |
| Malignant melanoma | 4.7 | (1.3, 16.5) | 0.0% | 0.0% |
| Eating disorders | 4.5 | (3.9, 5.1) | 1.2% | 0.3% |
| Atrial fibrillation | 4.5 | (1.8, 10.9) | 0.0% | 0.0% |
| Sleep disorders | 4.1 | (3.7, 4.4) | 2.6% | 0.7% |
| Fibromyalgia | 3.9 | (1.8, 8.4) | 0.0% | 0.0% |
| Epilepsy | 3.7 | (3.3, 4.1) | 1.5% | 0.4% |
| Chronic fatigue syndrome | 3.5 | (2.8, 4.5) | 0.3% | 0.1% |
| Menopause | 3.5 | (1.8, 6.9) | 0.0% | 0.0% |
| Avoidant/restrictive food intake disorder | 3.5 | (2.3, 5.3) | 0.1% | 0.0% |
| Non-infectious hepatitis | 3.4 | (1.5, 7.7) | 0.0% | 0.0% |

| Obstructive sleep apnea | 3.4 | (3.0, 3.9) | 1.4% | 0.4% |
| --- | --- | --- | --- | --- |
| Hypercoagulable syndrome | 3.3 | (1.9, 5.9) | 0.1% | 0.0% |
| Peripheral arterial disease | 3.3 | (2.5, 4.3) | 0.3% | 0.1% |
| Migraine | 3.2 | (3.0, 3.5) | 3.0% | 0.9% |
| Hypertension | 3.1 | (2.6, 3.8) | 0.5% | 0.2% |
| Kidney stones | 3.0 | (2.1, 4.3) | 0.2% | 0.1% |
| Pancreatitis | 2.8 | (1.3, 5.9) | 0.0% | 0.0% |
| Hyperlipidemia | 2.6 | (2.3, 2.8) | 1.9% | 0.8% |
| Low vision and blindness | 2.5 | (2.3, 2.7) | 4.2% | 1.7% |
| Ventricular arrhythmia | 2.4 | (1.7, 3.5) | 0.2% | 0.1% |
| Leukemia/myeloma | 2.4 | (1.5, 3.8) | 0.1% | 0.0% |
| Brain cancer | 2.3 | (1.4, 4.0) | 0.1% | 0.0% |
| Cerebrovascular disease | 2.3 | (1.6, 3.2) | 0.2% | 0.1% |
| Chronic thyroid disorders | 2.3 | (2.0, 2.6) | 1.3% | 0.6% |
| Osteoarthritis | 2.2 | (1.4, 3.5) | 0.1% | 0.0% |
| Obesity | 2.2 | (2.1, 2.3) | 10.1% | 4.8% |
| Peptic ulcer disease | 2.2 | (1.2, 4.1) | 0.0% | 0.0% |
| Allergic rhinitis | 2.1 | (2.1, 2.2) | 19.3% | 10.1% |
| Hemophilia/congenital coagulopathies | 2.1 | (1.3, 3.4) | 0.1% | 0.0% |
| Rheumatoid arthritis | 2.0 | (1.4, 3.0) | 0.1% | 0.1% |
| Diabetes mellitus | 2.0 | (1.7, 2.5) | 0.5% | 0.3% |
| Cholelithiasis/cholecystitis | 2.0 | (1.1, 3.6) | 0.1% | 0.0% |

Abbreviations: ADHD, attention-deficit/hyperactivity disorder; CI, confidence interval; OR, odds ratio

**^a^** Includes all OR values >2.0 (ranked; all *P*=0.000)

# Supplementary Table 6. Ratios with 95% Confidence Interval for Co-Existing Diagnoses Among Children with AuDHD

|  |  |  | **Cohort Prevalence (%)** | |
| --- | --- | --- | --- | --- |
| **Co-Existing Diagnoses** | **OR^a^** | **95 CI% (lower, upper)** | **AuDHD** | **Comparison** |
| Alzheimer's dementia | 162.8 | (10.2, 2603.7) | 0.0% | 0.0% |
| Disruptive childhood disorders | 44.4 | (39.8, 49.4) | 18.0% | 0.5% |
| Schizophrenia | 31.0 | (20.3, 47.1) | 1.0% | 0.0% |
| Non-Alzheimer’s dementia | 28.5 | (15.0, 54.2) | 0.4% | 0.0% |
| Cystic fibrosis | 25.7 | (14.6, 45.3) | 0.5% | 0.0% |
| Bipolar disorder | 24.3 | (18.7, 31.7) | 2.4% | 0.1% |
| Specific personality disorders | 20.3 | (13.1, 31.3) | 0.9% | 0.0% |
| Anxiety | 17.9 | (16.5, 19.3) | 46.9% | 4.7% |
| Alcohol use disorder | 15.5 | (9.9, 24.3) | 0.8% | 0.1% |
| Suicidal ideation | 13.2 | (10.3, 17.0) | 2.6% | 0.2% |
| Fibromyalgia | 12.9 | (4.0, 41.7) | 0.1% | 0.0% |
| Epilepsy | 12.2 | (10.2, 14.6) | 4.9% | 0.4% |
| Avoidant/restrictive food intake disorder | 12.1 | (6.5, 22.3) | 0.4% | 0.0% |
| Gender dysphoria | 11.3 | (7.4, 17.3) | 0.9% | 0.1% |
| Depression | 9.8 | (8.8, 10.8) | 18.6% | 2.3% |
| Sleep disorders | 9.0 | (7.6, 10.7) | 5.7% | 0.7% |
| Hypercoagulable syndrome | 8.4 | (3.1, 22.8) | 0.2% | 0.0% |
| Chronic renal failure | 7.9 | (4.0, 15.4) | 0.3% | 0.0% |
| Obstructive sleep apnea | 7.5 | (6.0, 9.5) | 2.9% | 0.4% |
| Eating disorders | 7.4 | (5.6, 9.7) | 2.0% | 0.3% |
| Psychiatric disorders related to medical conditions | 7.3 | (5.6, 9.4) | 2.3% | 0.3% |
| Post-traumatic stress disorder | 6.6 | (4.5, 9.8) | 1.0% | 0.2% |
| Peptic ulcer disease | 5.3 | (1.7, 16.6) | 0.1% | 0.0% |
| Connective tissue disorders | 5.2 | (2.3, 11.7) | 0.2% | 0.0% |
| Down's syndrome | 4.9 | (2.6, 9.2) | 0.4% | 0.1% |
| Inflammatory bowel disease | 4.3 | (2.3, 8.1) | 0.4% | 0.1% |

| Hypertension | 4.3 | (2.7, 6.9) | 0.7% | 0.2% |
| --- | --- | --- | --- | --- |
| Cerebrovascular disease | 3.9 | (1.8, 8.2) | 0.3% | 0.1% |
| Hyperlipidemia | 3.8 | (3.0, 4.8) | 2.8% | 0.8% |
| Peripheral arterial disease | 3.6 | (1.8, 7.3) | 0.3% | 0.1% |
| Migraine | 3.5 | (2.8, 4.4) | 3.2% | 0.9% |
| Obesity | 3.4 | (3.1, 3.8) | 14.8% | 4.8% |
| Chronic thyroid disorders | 3.3 | (2.5, 4.4) | 1.8% | 0.6% |
| Chronic fatigue syndrome | 3.2 | (1.6, 6.5) | 0.3% | 0.1% |
| Periodontal disease | 2.7 | (1.5, 4.9) | 0.4% | 0.2% |
| Allergic rhinitis | 2.7 | (2.5, 3.0) | 23.4% | 10.1% |
| Substance related disorders | 2.5 | (1.5, 4.4) | 0.5% | 0.2% |
| Congenital heart disease | 2.5 | (1.9, 3.1) | 2.6% | 1.1% |
| Diabetes mellitus | 2.3 | (1.4, 3.8) | 0.6% | 0.3% |
| Asthma | 2.2 | (2.0, 2.5) | 12.6% | 6.1% |

Abbreviations: AuDHD, co-existing autism spectrum disorder and attention-deficit/hyperactivity disorder; CI, confidence interval; OR, odds ratio

**^a^** Includes all OR values >2.0 (ranked; all *P*=0.000)
